# Untargeted Metabolomics Reveals Organ-Specific Metabolites Associated with Antioxidant and Anti-Inflammatory Activities in Finger Citron (*Citrus medica* L. var. *sarcodactylis*)

**DOI:** 10.3390/metabo16080555

**Published:** 2026-08-06

**Authors:** Xi Yi, Xin Zeng, Yu Zhang, Junxi Zhao, Lin Zeng, Wei Xiang, Jianwei Wang

**Affiliations:** 1 College of Traditional Chinese Medicine, Chongqing Medical University, Chongqing 400016, China; yeexien@163.com; 2 Chongqing Key Laboratory of Traditional Chinese Medicine for Prevention and Cure of Metabolic Diseases, Chongqing University of Chinese Medicine, Chongqing 402760, China; zeng_xin928@163.com (X.Z.); 18623165015@163.com (Y.Z.); 18623372142@163.com (J.Z.); 13350379627@163.com (L.Z.)

**Keywords:** *Citrus medica* L. var. *sarcodactylis*, finger citron, untargeted metabolomics, organ-specific metabolites, non-medicinal parts, antioxidant activity, anti-inflammatory activity

## Abstract

Background: Finger citron (*Citrus medica* L. var. *sarcodactylis* Swingle, CM) is an important edible and medicinal plant. Owing to its diverse pharmacological properties, its fruit has long served as a traditional Chinese medicine and has increasingly been developed for functional food applications. However, its non-fruit parts, including leaves, branches, and roots, remain underutilized. Methods: In this study, CM fruit (CMF), leaves (CML), roots (CMR), and branches (CMB) were selected to systematically compare their small-molecule metabolic profiles and antioxidant and anti-inflammatory activities. Untargeted metabolomics was conducted based on ultra-performance liquid chromatography–tandem mass spectrometry (UPLC-MS/MS). Meanwhile, antioxidant capacity was evaluated using 2,2-diphenyl-1-picrylhydrazyl (DPPH) and 2,2′-azino-bis (3-ethylbenzothiazoline-6-sulfonic acid) (ABTS) radical scavenging assays, and anti-inflammatory activity was assessed using a lipopolysaccharide (LPS)-induced RAW 264.7 macrophage inflammation model. Results: Metabolomic analysis revealed 826 differential metabolites across the four CM parts (CMs), reflecting pronounced organ-specific metabolic features. According to Kyoto Encyclopedia of Genes and Genomes (KEGG) enrichment analysis, the variations in metabolite profiles across the four CMs were primarily linked to amino acid metabolism, phenylalanine metabolism, and unsaturated fatty acid metabolism. In the antioxidant assays, the DPPH radical scavenging activity at 2 mg/mL followed the order CML (78.37 ± 7.29%), CMR (73.30 ± 2.68%), CMB (40.71 ± 0.16%), and CMF (36.67 ± 1.67%), whereas ABTS radical scavenging activity followed the order CMR (51.36 ± 2.17%), CML (48.30 ± 1.64%), CMB (45.23 ± 1.72%), and CMF (18.48 ± 0.22%). In the LPS-stimulated RAW 264.7 macrophage model, at 0.4 mg/mL, inhibition of nitric oxide (NO) production followed the order CML (88.18 ± 4.02%), CMB (87.21 ± 3.02%), CMF (68.05 ± 2.54%), and CMR (67.21 ± 9.12%). Correlation analysis further suggested that features putatively annotated as minecoside, maltotetraose, and (+)-catechin may be associated with antioxidant or anti-inflammatory activities. Conclusions: The study revealed differences in metabolite composition and bioactivities among different CMs, providing an experimental basis for the development and utilization of non-fruit parts, especially leaves, and for further investigation of their bioactive constituents.

## 1. Introduction

Finger citron (*Citrus medica* L. var. *sarcodactylis* Swingle, CM) is an edible and medicinal plant belonging to the genus *Citrus* of the family Rutaceae. It is widely cultivated in Sichuan, Fujian, Guangdong, Guangxi and other regions of China and is recognized as an important plant resource with medicinal, edible, and ornamental value [1]. Among its parts, CM fruit (CMF), the mature fruit of the plant, is the principal medicinal part and has been the main focus of traditional Chinese medicine use, modern pharmacological research, and functional food development. According to traditional Chinese medicine theory, CMF is considered to soothe the liver and regulate qi, harmonize the stomach and relieve pain, and dry dampness and resolve phlegm. Therefore, it has traditionally been used for conditions associated with liver-stomach qi stagnation, as well as cough with excessive phlegm [2,3]. Recent pharmacological evidence indicates that CMF and its extracts exhibit multiple bioactivities, including antioxidant [4], anti-inflammatory [5], antitumor [6], hypolipidemic [7], hypoglycemic [8], and gut microbiota-modulatory activities [9]. In addition to its medicinal uses, CMF is also commonly used in tea infusions, soups, candied products, and spice processing, demonstrating high application value in food, health-promoting products, and daily dietary consumption [10,11]. The traditional efficacy and pharmacological activities of CMF are closely related to a variety of active small molecules, particularly flavonoids, phenolic acids, coumarins, and volatile constituents of essential oils [11,12].

During the cultivation, pruning, harvesting, and processing of CM, its non-fruit parts, including leaves, branches, and roots, remain underutilized and are often discarded directly as agricultural waste, thereby preventing the full value of this plant resource from being realized [13,14]. Previous studies have indicated that the non-fruit parts of CM also possess bioactive potential. The methanolic extract of CM leaves (CML) and its different polarity fractions exhibited antifungal activity against *Candida albicans*, which may be associated with flavonoids and phenolic acids [15]. Furthermore, studies on CM stem bark and root bark have led to the characterization of citrumedin-B, a novel coumarin, as well as several known compounds with anti-inflammatory activity [16], suggesting that the non-fruit parts of CM may also be rich in bioactive secondary metabolites. However, current studies are mainly limited to a single plant part, a single biological activity, or the isolation and identification of a few compounds, and systematic comparative metabolomics studies coupled with bioactivity correlation analysis among CMF, CML, CMB, and CMR remain lacking. Therefore, from the perspective of comprehensive plant resource utilization, systematically elucidating the differences in small-molecule metabolite profiles and potential bioactivities among different CM parts (CMs) would improve our understanding of the overall value of CM resources and provide a scientific basis for the rational development and utilization of its non-fruit parts.

To clarify the metabolic and functional differences among different CM parts, CMF, CML, CMR, and CMB were selected for comparative analysis. Their small-molecule metabolites were profiled using ultra-performance liquid chromatography–tandem mass spectrometry (UPLC-MS/MS). Meanwhile, the antioxidant and anti-inflammatory properties of extracts from CMs were evaluated using 2,2-diphenyl-1-picrylhydrazyl (DPPH) and 2,2′-azino-bis (3-ethylbenzothiazoline-6-sulfonic acid) (ABTS) radical scavenging assays and a lipopolysaccharide (LPS)-induced RAW 264.7 macrophage inflammation model. We hypothesized that different CMs may exhibit distinct metabolite accumulation patterns and antioxidant and anti-inflammatory activities because of their differing physiological functions. Therefore, we employed UPLC–MS/MS-based untargeted metabolomics combined with in vitro bioactivity assays to compare the metabolic profiles and bioactivities of the four CMs and to identify candidate metabolites potentially associated with the observed activity differences. The findings will help elucidate the chemical composition and application potential of the non-fruit parts of CM and provide a foundation for further validation of bioactive constituents, quality evaluation, and comprehensive utilization of CM plant resources.

## 2. Materials and Methods

### 2.1. Chemicals and Materials

Acetonitrile and ethanol of LC-MS grade were purchased from ANPEL Laboratory Technologies (Shanghai) Inc. (Shanghai, China). Ultrapure water was prepared using a Milli-Q water purification system (MilliporeSigma, Burlington, MA, USA). Reagents used for antioxidant activity assays, including DPPH, ABTS, potassium persulfate, and butylated hydroxytoluene, were purchased from Shanghai Macklin Biochemical Co., Ltd. (Shanghai, China). The RAW 264.7 murine macrophage cell line (Cat. No. SCSP-5036) was obtained from the National Collection of Authenticated Cell Cultures, Chinese Academy of Sciences (Shanghai, China). High-glucose Dulbecco’s modified Eagle’s medium (DMEM) used for cell culture was obtained from Thermo Fisher Scientific (Waltham, MA, USA), while lipopolysaccharide (LPS) was provided by Sigma-Aldrich (St. Louis, MO, USA). Fetal bovine serum (FBS) was obtained from Suzhou Haixing Bioscience Co., Ltd. (Suzhou, China). Penicillin–streptomycin solution was supplied by Beyotime Biotechnology (Shanghai, China), whereas the CCK-8 assay kit and ELISA kits were provided by Heyuan Liji (Shanghai, China) Biotechnology Co., Ltd. (Shanghai, China), and Jiangsu Meimian Industrial Co., Ltd. (Yancheng, China), respectively.

### 2.2. Sample Preparation and Extraction

Fresh leaves (CML), roots (CMR), fruits (CMF), and branches (CMB) of CM plants were collected from Jinshan Town, Dazu District, Chongqing, China (29°64′ N, 105°91′ E). Three independent *Citrus medica* plants were selected for biological sampling. Fruits, leaves, roots, and branches were separately collected from each individual plant. Thus, each CM part included three independent biological replicates (*n* = 3), with each replicate derived from an individual plant. Each biological sample was independently subjected to extraction, sample preparation, and UPLC–MS/MS analysis. All biological samples were analyzed in a randomized order. After collection, the samples were rapidly snap-frozen in liquid nitrogen and then kept at −80 °C until further processing. Before extraction, the frozen materials were thawed on ice at 4 °C and processed without delay. Fresh tissue from each sample was weighed at 5.0 g and mixed with 50 mL of 70% (*v*/*v*) aqueous ethanol for extraction. The suspension was treated ultrasonically for 30 min at 300 W under controlled temperature conditions of 25 ± 2 °C, followed by centrifugation at 6000 rpm for 10 min. After removal of the solid residue, the recovered extract was evaporated under reduced pressure until dry and reconstituted in 10 mL of 50% (*v*/*v*) aqueous acetonitrile, corresponding to a fresh-tissue equivalent of 0.5 g/mL. The extraction yield was calculated based on the ratio of dried extract weight to the initial fresh tissue weight, and the corresponding extraction yields of different CMs are summarized in Table S1. The samples were then vortex-mixed and centrifuged at 12,000 rpm and 4 °C for 15 min. The clarified solutions were filtered through a 0.22 μm membrane before UPLC–MS/MS analysis. All extracts used for metabolomic analysis were normalized based on the equivalent fresh tissue concentration (0.5 g/mL) to ensure comparable sample input among different CMs. For bioactivity assays, dried extracts were reconstituted and tested at equivalent extract concentrations to evaluate the intrinsic biological activity of each CM extract.

### 2.3. UPLC–MS/MS Analysis

Metabolomic profiling of the prepared extracts was conducted using a UPLC system coupled to a Q Exactive HF-X Orbitrap MS/MS platform (Thermo Fisher Scientific, Waltham, MA, USA). Chromatographic separation was achieved on an ACQUITY UPLC HSS T3 column (100 mm × 2.1 mm, 1.8 μm; Waters, Milford, MA, USA) with water and acetonitrile, both containing 0.1% formic acid, as the mobile phases. An injection volume of 2 μL was used to minimize the amount of organic solvent introduced onto the column. After injection, the mobile phase was maintained at 0% B for 1 min, which was intended to facilitate the refocusing of sufficiently retained analytes at the head of the HSS T3 column and reduce the potential solvent-mismatch effect caused by the 50% aqueous acetonitrile sample diluent. Data were collected in Full MS/dd-MS^2^ mode under positive/negative ion switching, with a full-scan range of *m*/*z* 70–1050. Detailed chromatographic and mass spectrometric parameters are provided in Table S2.

### 2.4. Data Processing, Metabolite Annotation, and Differential Metabolite Screening

Raw UPLC–MS/MS data were acquired using Xcalibur 4.1 software (Thermo Fisher Scientific, Waltham, MA, USA). Data preprocessing, including baseline filtering, peak detection, peak matching, retention-time correction, peak alignment, and generation of the peak-intensity matrix, was performed using Progenesis QI software, version 3.0.7927 (Waters Corporation, Milford, MA, USA). Features with zero values in more than 80% of all samples were removed, and the remaining data were normalized using the MetNormalizer package (v1.3.02) in R. QC samples were prepared by pooling equal volumes of all biological sample extracts and were used to assess analytical stability. Features with QC relative standard deviation (RSD) values > 30% were excluded. Metabolite features with available MS/MS spectra were putatively annotated (MSI Level 2 confidence) based on spectral matching against HMDB and METLIN databases. Candidate annotations were evaluated based on accurate molecular mass (mass error within 10 ppm) and MS/MS fragmentation patterns. MS/MS spectral similarity was assessed using a library-matching score ranging from 0 to 100, with higher scores indicating greater annotation confidence. HMDB, KEGG Compound, and LIPID MAPS databases were subsequently used for metabolite information integration, KEGG identifier assignment, and pathway annotation. KEGG pathway annotation was performed by mapping annotated metabolites to the corresponding entries in the publicly available KEGG Compound database. Principal component analysis (PCA) and partial least-squares discriminant analysis (PLS-DA) were performed using the ropls package (version 1.32.0) in R. PCA was used as an unsupervised method to visualize overall metabolic variation among samples, while PLS-DA was applied as a supervised method for group discrimination. The reliability of the PLS-DA model was evaluated using cumulative R^2^Y and Q^2^ values, together with a 200-permutation test. Statistical differences among the four CM parts were assessed using one-way analysis of variance (ANOVA), followed by Tukey’s multiple-comparisons test. The assumptions of normality and homogeneity of variance were evaluated before ANOVA analysis. Differential metabolites were screened by integrating multivariate statistical analysis (VIP > 1), univariate statistical analysis (*p* < 0.05), and fold-change criteria (FC > 1.5 or FC < 0.67). Because authentic standards were not available for all annotated metabolites, retention-time matching against reference standards and targeted quantitative validation were not performed for every candidate metabolite. Therefore, the metabolites reported in this study require further confirmation using authentic standards. Detailed information regarding data processing, metabolite annotation, and differential-metabolite screening parameters is provided in Table S3.

### 2.5. In Vitro Antioxidant Activity Assays

The 70% ethanol extracts were used as stock solutions for the DPPH and ABTS radical scavenging assays and were diluted to the required working concentrations with 70% ethanol. The sample concentrations in the antioxidant assays were expressed as dry extract equivalents. The antioxidant capacities of the extracts were evaluated using DPPH and ABTS radical scavenging assays according to previously reported procedures with minor modifications [17]. For both assays, extract solutions were prepared at concentrations of 0, 0.25, 0.5, 1.0, and 2.0 mg/mL, while BHT was used as the positive control at the same concentrations. In the DPPH assay, the reaction mixtures were incubated for 30 min in the dark, and the absorbance was recorded at 517 nm. For the ABTS assay, the ABTS•+ working solution was prepared from ABTS and potassium persulfate and adjusted to an absorbance of 0.70 ± 0.02 at 734 nm before use. After reaction with the extracts for 5 min, the absorbance was measured at 734 nm. All determinations were conducted in triplicate, and radical scavenging activities were calculated as described in the reported method [17]. Detailed experimental conditions are provided in Table S4.

### 2.6. Anti-Inflammatory Activity Evaluation

For cell-based assays, the ethanol extracts were concentrated, lyophilized, dissolved in DMSO, and further diluted with culture medium to the desired concentrations. The DMSO content in all treatment solutions was kept below 0.1% (*v*/*v*), and the prepared solutions were sterilized by filtration through a 0.22 μm membrane before use. RAW 264.7 murine macrophages were maintained in high-glucose DMEM containing 10% FBS and 1% penicillin–streptomycin under standard culture conditions. An inflammatory response was induced by stimulation with lipopolysaccharide (LPS, 1 μg/mL) for 24 h. Untreated cells were used as the control group, whereas cells exposed to LPS alone represented the model group. Cell viability was assessed using the CCK-8 assay after treatment with different concentrations of the extracts [18]. Nitric oxide (NO) production was evaluated by determining nitrite accumulation in the culture supernatants using Griess reagent. The secretion levels of TNF-α, IL-6, and IL-1β were quantified using ELISA kits following the manufacturers’ protocols. All assays were performed in triplicate, and detailed experimental conditions are provided in Table S5.

### 2.7. Statistical Analysis

Data are presented as the mean ± standard deviation (SD) based on at least three independent biological replicates. IC_50_ values were estimated by nonlinear curve fitting in GraphPad Prism 10.1.2. Associations between metabolite levels and IC_50_ values related to biological activities were evaluated using Spearman’s rank correlation. Differences among groups were assessed by one-way analysis of variance (ANOVA), followed by Tukey’s multiple-comparisons test. Statistical significance was defined as *p* < 0.05.

## 3. Results

### 3.1. Analysis of Differential Small-Molecule Metabolites in Different Parts of Finger Citron

#### 3.1.1. Putative Annotation and Classification of Metabolite Features

**Figure 1:**
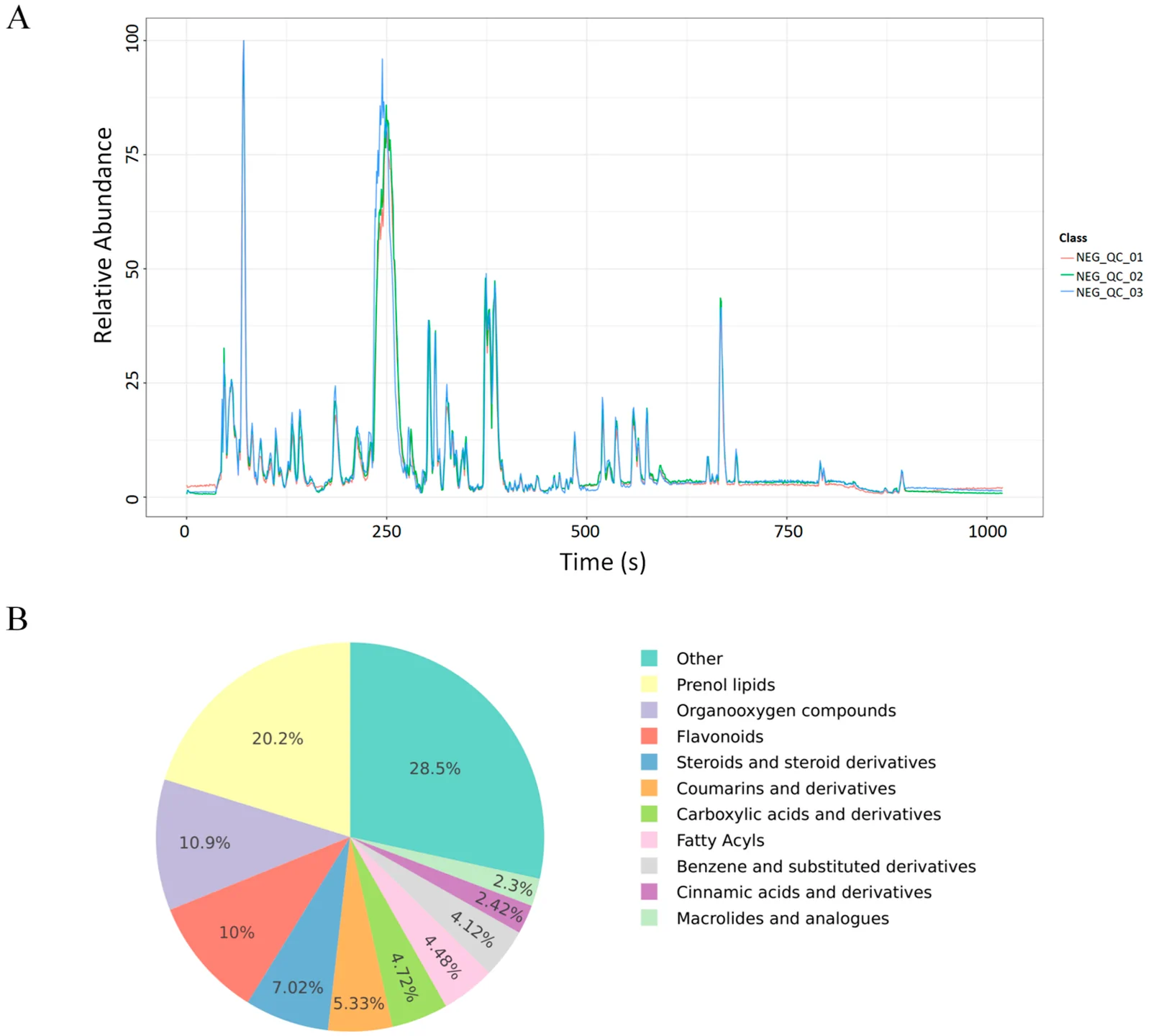
(**A**) Total ion chromatograms of metabolites from different parts of *Citrus medica* L. var. sarcodactylis detected by a UPLC system coupled with a Q Exactive HF-X Orbitrap MS/MS platform; (**B**) Classification of detected metabolites.

To systematically characterize the small-molecule metabolite composition of different CMs, untargeted metabolomic analysis of CMR, CML, CMB, and CMF was performed using a UPLC system coupled with a Q Exactive HF-X Orbitrap MS/MS platform. As shown in Figure 1A, the chromatographic profiles of the quality control (QC) samples were highly consistent, with closely matched retention times and peak signal intensities, supporting the reproducibility of the analytical procedure and the reliability of the acquired data.

A total of 930 annotated metabolites were obtained before statistical filtering. Using VIP > 1, *p* < 0.05, and FC > 1.5 or FC < 0.67 as the selection thresholds, 826 differential metabolites were subsequently screened from the four CMs and assigned to 11 chemical classes (Figure 1B, Table S6). Excluding those assigned to “Others”, prenol lipids constituted the most abundant category (167 metabolites, 20.2%), followed by organooxygen compounds (90, 10.9%), flavonoids (83, 10.0%), steroids and steroid derivatives (58, 7.02%), coumarins and derivatives (44, 5.33%), and carboxylic acids and derivatives (39, 4.72%). Additionally, fatty acyls (37, 4.48%), benzene and substituted derivatives (34, 4.12%), cinnamic acids and derivatives (20, 2.42%), and macrolides and analogues (19, 2.30%) were also detected.

#### 3.1.2. Multivariate Statistical Analysis and Sample Clustering

**Figure 2:**
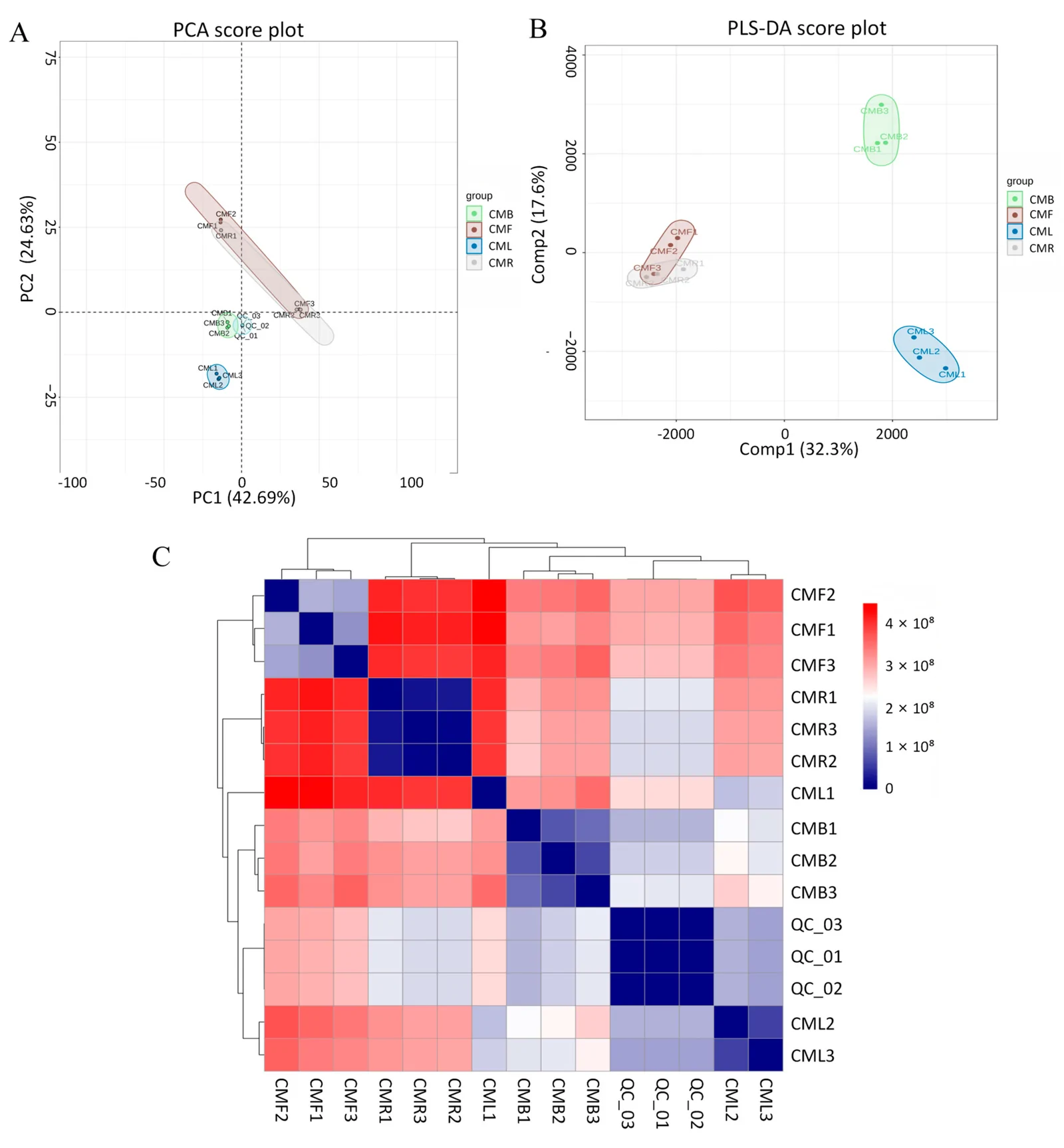
(**A**) PCA score plot of biological samples and pooled quality control (QC) samples; (**B**) PLS-DA score plot; (**C**) Hierarchical clustering heatmap of differential metabolites.

To evaluate the overall metabolic variation among different CMs, principal component analysis (PCA), an unsupervised multivariate statistical approach, was initially performed. As shown in Figure 2A, the first two principal components (PC1 and PC2) explained 43.55% and 25.00% of the total variance, respectively, together accounting for 68.55% of the overall variation. Biological replicates from the same CM part showed relatively close clustering, indicating good experimental consistency and reproducibility. The pooled QC samples were included in the PCA score plot together with biological samples and showed tight clustering, indicating good stability and reproducibility of the LC-MS/MS analytical procedure. The PCA score plot revealed that CMF and CMR samples exhibited partial overlap, suggesting similarities in their metabolic profiles. Subsequently, partial least-squares discriminant analysis (PLS-DA), a supervised multivariate statistical method, was performed to further evaluate metabolic differences among different CMs (Figure 2B). The first two components of the PLS-DA model (Comp1 and Comp2) accounted for 32.3% and 17.6% of the total variation, respectively, together explaining 49.9%. The close grouping of biological replicates from the same sample type suggested high experimental consistency and satisfactory reproducibility. The score plot revealed that CMF and CMR occupied neighboring positions in the left portion and partly overlapped, suggesting a certain similarity in their metabolic characteristics. In contrast, the CMB and CML groups were distributed on the right side and separated primarily along Comp2, indicating marked differences from the CMF and CMR groups. The reliability of the PLS-DA model was further evaluated based on cumulative R^2^Y and Q^2^ values together with a 200-permutation test. The PLS-DA model constructed from the four CMs showed satisfactory explanatory and predictive performance, with R^2^Y(cum) = 0.654 and Q^2^(cum) = 0.372 (Table S7). Furthermore, the permutation test showed that the Q^2^ values obtained from the permuted models were lower than that of the original model, and the Q^2^ regression line intersected the Y-axis below zero, indicating that the PLS-DA model was not overfitted (Figure S1). Hierarchical clustering heatmap analysis further confirmed these results (Figure 2C). The CMB group showed markedly lower correlations with the other groups and formed an independent cluster, whereas the CMF and CMR groups clustered closely together and showed some degree of correlation with the CML group.

#### 3.1.3. Hierarchical Clustering of Differential Metabolites and Tissue Specificity

**Figure 3:**
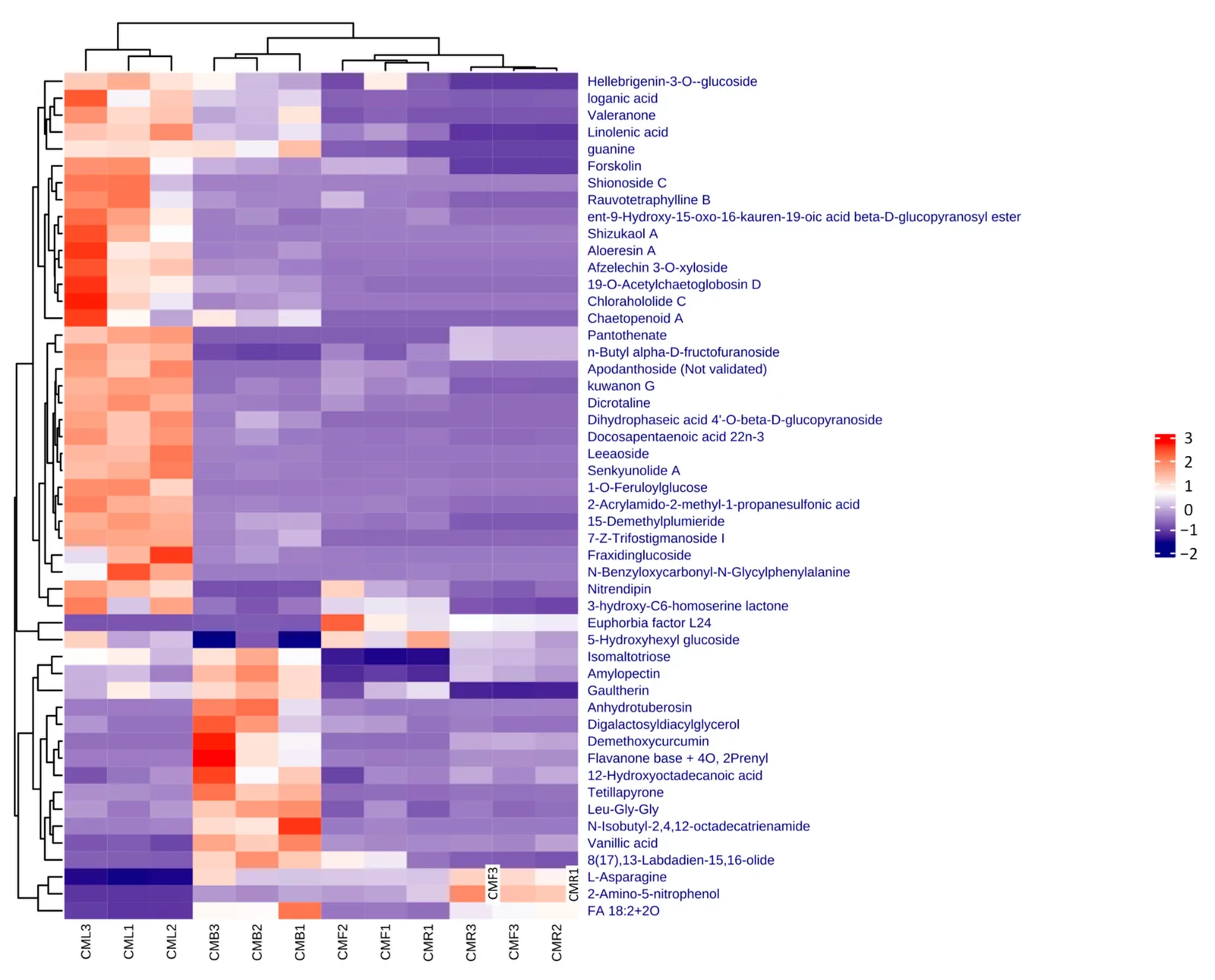
Heatmap of differential metabolites in CMR, CML, CMB, and CMF.

Hierarchical clustering analysis showed that the differential metabolites in different CMs exhibited distinct tissue-specific distribution patterns (Figure 3). Samples from the same part showed good clustering consistency, whereas the relative abundance patterns differed markedly among different parts. Based on the putative annotations, several differential metabolite features were relatively enriched in CML, including features putatively annotated as terpenoids (forskolin and shizukaol A), flavonoid and polyphenolic compounds (kuwanon G and fraxidinglucoside), alkaloids (rauvotetraphylline B), and unsaturated fatty acids (linolenic acid). In CMB, the enriched metabolites mainly included saccharides (amylopectin and isomaltotriose), phenolic compounds (demethoxycurcumin and vanillic acid), and lipid metabolites (12-hydroxyoctadecanoic acid). In contrast, relatively fewer differential metabolites were enriched in CMR and CMF. CMR was characterized mainly by the enrichment of L-asparagine and oxidized fatty acids (FA 18:2+2O), whereas CMF was characterized by the enrichment of Euphorbia factor L24 and 3-hydroxy-C6-homoserine lactone. These results indicate significant differences in metabolite accumulation among different CM tissues, characterized by smaller within-group variation and larger between-group variation, with leaves and branches showing more prominent tissue-specific metabolic features.

#### 3.1.4. Pathway Enrichment Analysis of Differential Metabolites

**Figure 4:**
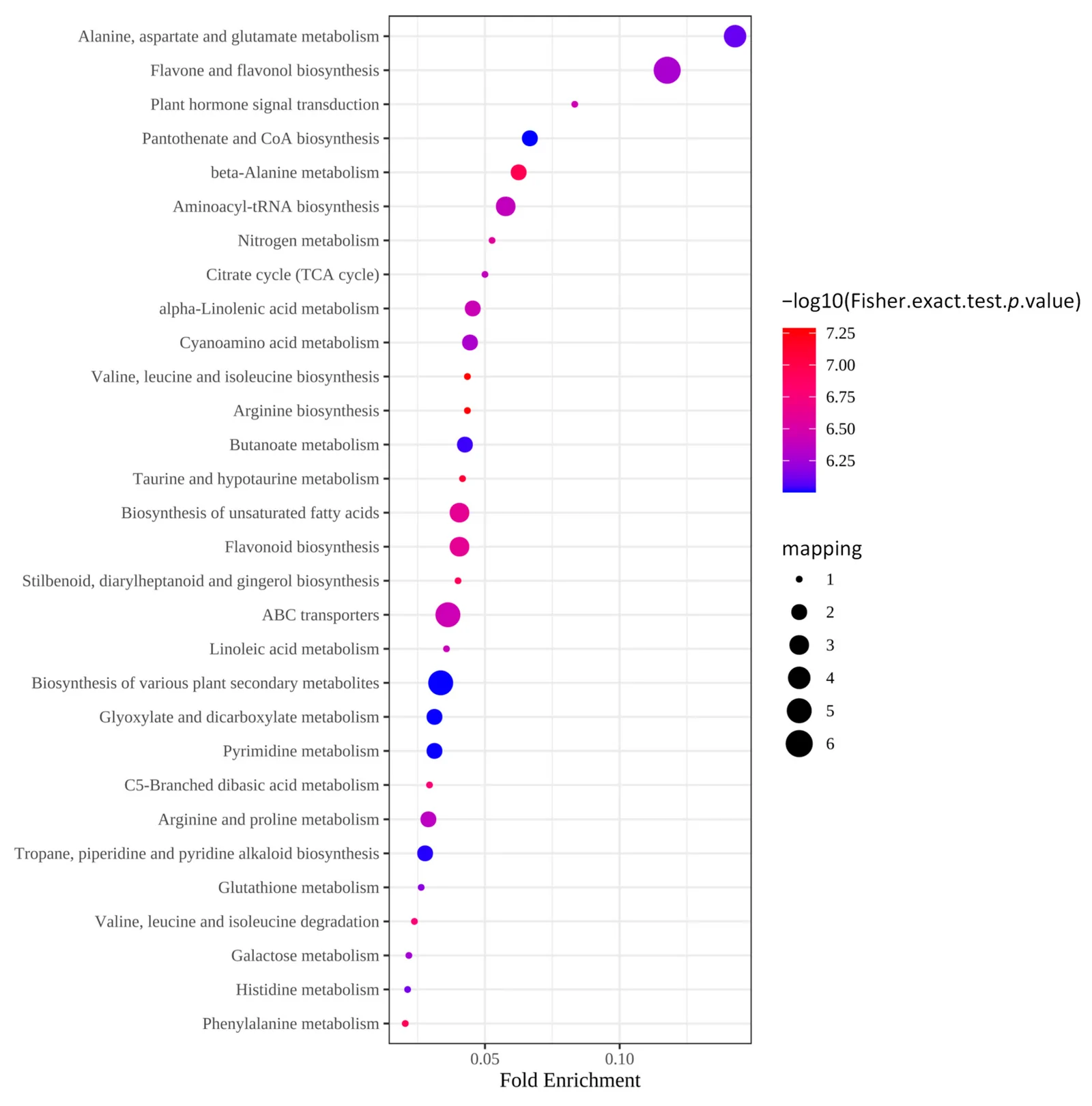
KEGG pathway enrichment analysis of differential metabolites in CMR, CML, CMB, and CMF.

To explore the biological functions associated with the differential metabolites, KEGG enrichment analysis was conducted, with the 30 pathways showing the highest significance selected based on *p* values (Figure 4). The five most significantly enriched pathways were arginine biosynthesis (ko00220, *p* = 5.15 × 10^−8^); valine, leucine, and isoleucine biosynthesis (ko00290, *p* = 5.15 × 10^−8^); taurine and hypotaurine metabolism (ko00430, *p* = 7.97 × 10^−8^); β-alanine metabolism (ko00410, *p* = 1.07 × 10^−7^); and phenylalanine metabolism (ko00360, *p* = 1.16 × 10^−7^). Among them, arginine biosynthesis and valine, leucine, and isoleucine biosynthesis showed the highest significance, both with a −log_10_(*p*) value of 7.29, suggesting that nitrogen metabolism and amino acid biosynthesis-related processes may be involved in the metabolic differentiation among different CMs.

In terms of differential metabolite coverage, the flavone and flavonol biosynthesis pathway (ko00944) contained the highest number of differential metabolites (six metabolites: luteolin, vitexin, apigenin, cynaroside, kaempferol-3-O-rutinoside, astragalin) among all enriched pathways. This was followed by ABC transporters (ko02010, five metabolites) and biosynthesis of various plant secondary metabolites (ko00999, five metabolites). Among the core differential metabolites, L-glutamic acid was involved in the largest number of pathways (12 pathways), including arginine biosynthesis, nitrogen metabolism, glutathione metabolism, and TCA cycle pathways. L-valine ranked second (six pathways), while **γ**-aminobutyric acid (four pathways), citric acid, and L-asparagine (three pathways each) were also involved in multiple pathways. Regarding lipid metabolism, biosynthesis of unsaturated fatty acids (ko01040), linoleic acid metabolism (ko00591), and α-linolenic acid metabolism (ko00592) were all significantly enriched. In addition, jasmonic acid was involved in both plant hormone signal transduction (ko04075) and α-linolenic acid metabolism, suggesting that lipid-derived signaling molecules may be involved in metabolic regulation.

### 3.2. Evaluation of Antioxidant Activity

**Figure 5:**
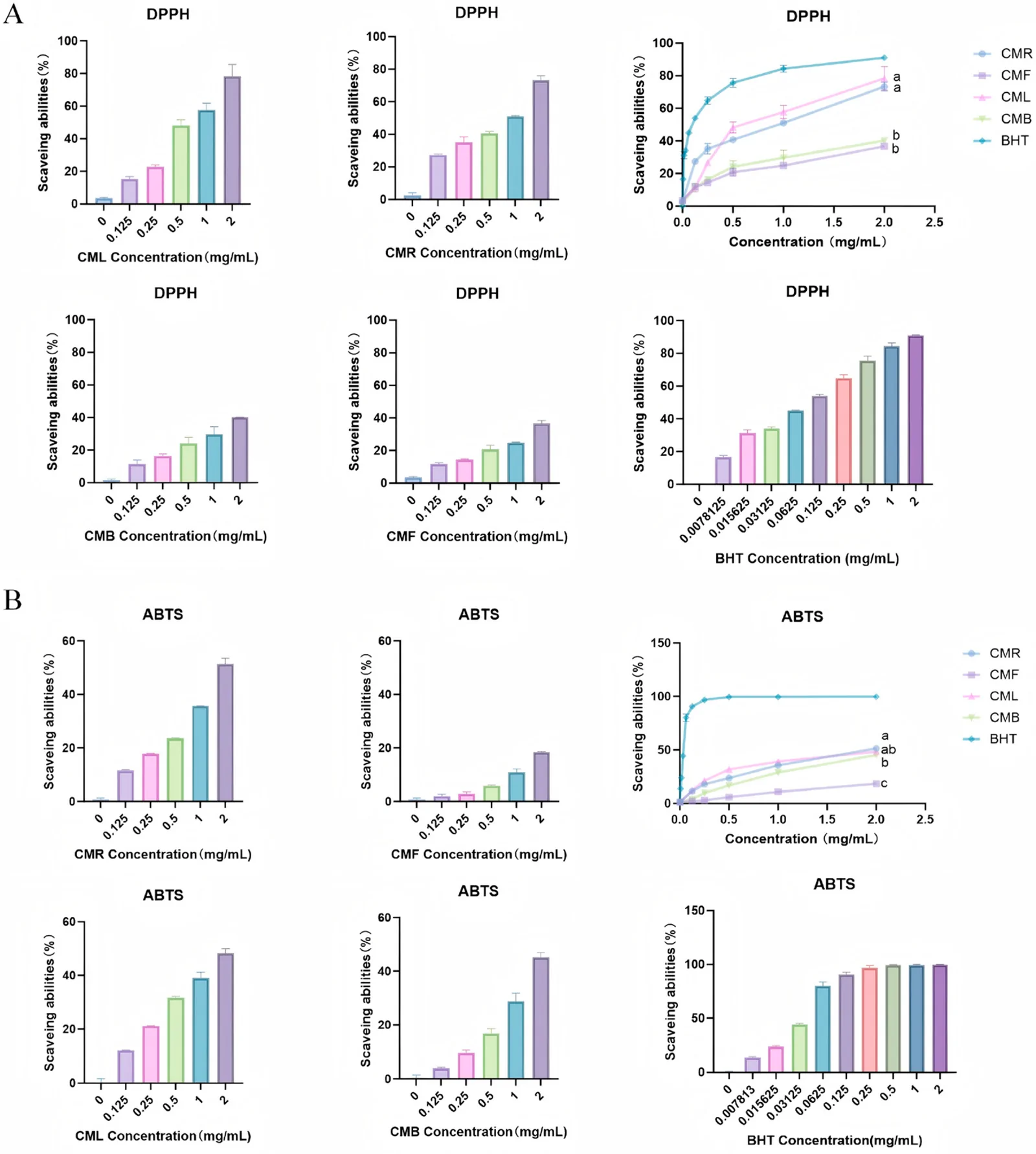
Antioxidant activities of *Citrus medica* L. var. *sarcodactylis*: (**A**) DPPH radical scavenging activity; (**B**) ABTS^+^ radical scavenging activity. Data are presented as mean ± standard deviation (SD) of three independent biological replicates. Different lowercase letters indicate significant differences among CM tissues (one-way ANOVA followed by Tukey’s multiple-comparisons test, *p* < 0.05).

The free radical-quenching capacities of extracts derived from different CMs were examined through DPPH and ABTS assays. Statistical comparisons among different tissues were performed using one-way ANOVA followed by Tukey’s multiple-comparisons test (*p* < 0.05). In the DPPH assay (Figure 5A), all extracts exhibited concentration-dependent increases in radical-scavenging activity, with CML and CMR presenting stronger antioxidant effects than the other groups. At 2 mg/mL, CML exhibited a DPPH radical-scavenging activity of 78.37 ± 7.29%, followed by CMR at 73.30 ± 2.68%, CMB at 40.71 ± 0.16% and CMF at 36.67 ± 1.67%. No significant difference was observed between CML and CMR or between CMB and CMF (*p* > 0.05). However, the scavenging activities of both CML and CMR were significantly higher than those of CMB and CMF (all *p* < 0.001). The IC_50_ values showed a similar pattern, with CML and CMR exhibiting relatively low IC_50_ values of 0.65 ± 0.08 mg/mL and 0.69 ± 0.05 mg/mL, respectively, with no significant difference between them (*p* > 0.05), while CMB and CMF exhibited IC_50_ values above 2 mg/mL. The ABTS assay results (Figure 5B) showed that, at 2 mg/mL, CMR exhibited the highest mean radical-scavenging activity (51.36 ± 2.17%), followed by CML (48.30 ± 1.64%), CMB (45.23 ± 1.72%), and CMF (18.48 ± 0.22%). No significant difference was observed between CMR and CML or between CML and CMB (*p* > 0.05). However, the ABTS radical-scavenging activity of CMR was significantly higher than those of CMB (*p* < 0.01) and CMF (*p* < 0.001). In addition, both CML and CMB exhibited significantly higher scavenging activities than CMF (both *p* < 0.001).

Taken together, CML and CMR exhibited consistently favorable antioxidant performance across the two assays. Nevertheless, the differences in activity rankings between the DPPH and ABTS assays suggest that the antioxidant properties of the different CM extracts may be influenced by differences in the abundance, composition, and reaction characteristics of their antioxidant constituents.

### 3.3. Evaluation of Anti-Inflammatory Activity

**Figure 6:**
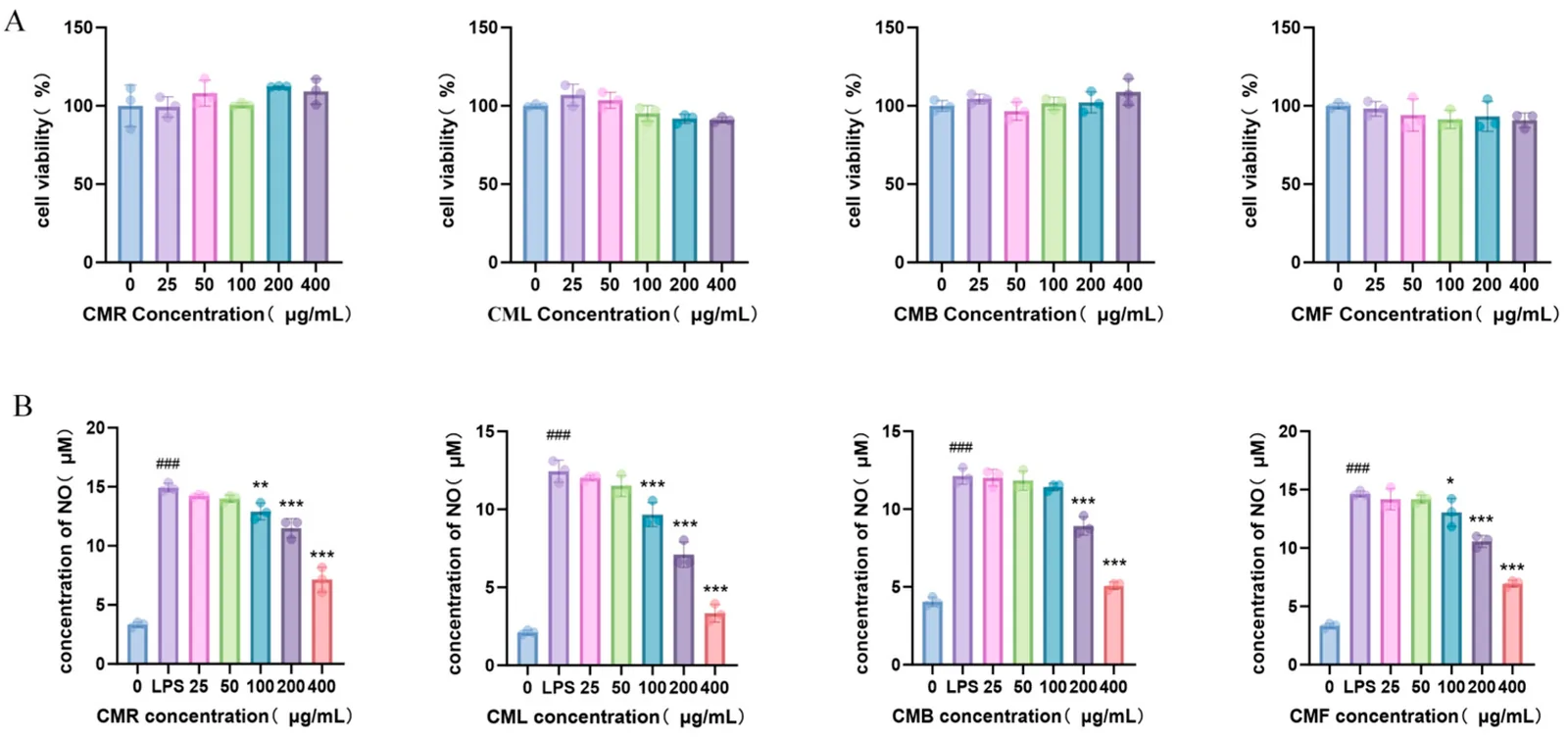
Protective effects of CMR, CML, CMB, and CMF against LPS-induced inflammatory injury in RAW264.7 cells. (**A**) Effect on RAW264.7 cell viability; (**B**) NO production. Results are presented as mean ± standard error (*n* = 3), and a *p*-value less than 0.05 was considered statistically significant (###, *p* < 0.001 compared with the control group, while * *p* < 0.05, ** *p* < 0.01 and *** *p* < 0.001 compared with the model group, respectively).

**Figure 7:**
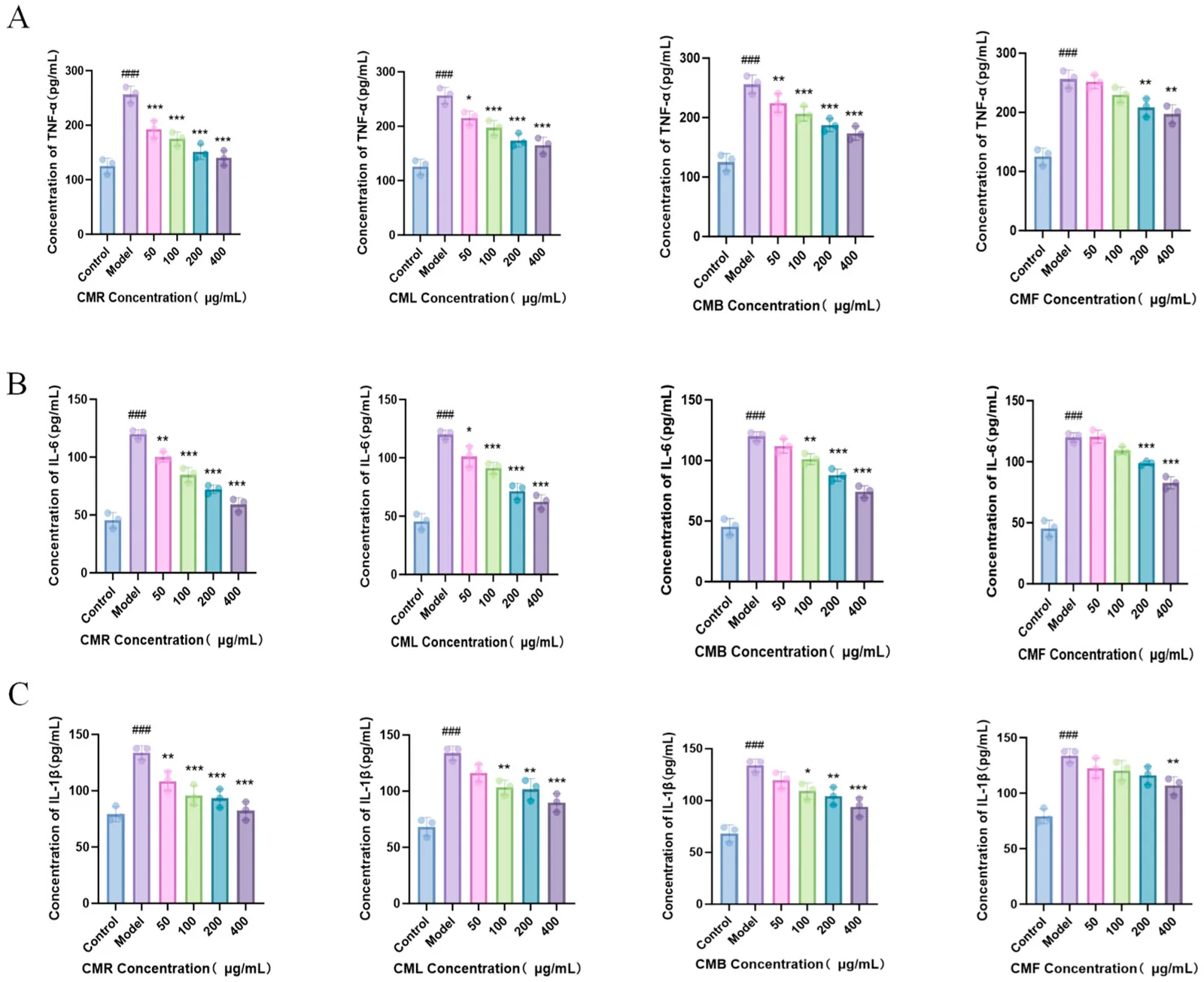
Effects of CMR, CML, CMB, and CMF on pro-inflammatory cytokines in LPS-induced RAW 264.7 cell model: (**A**) TNF-α, (**B**) IL-6, (**C**) IL-1β. Results are presented as mean ± standard error (*n* = 3), and a *p*-value less than 0.05 was considered statistically significant (###, *p* < 0.001 compared with the control group, while * *p* < 0.05, ** *p* < 0.01, and *** *p* < 0.001 compared with the model group, respectively).

LPS stimulation can activate RAW 264.7 macrophages and induce the expression of inducible nitric oxide synthase (iNOS), resulting in increased nitric oxide (NO) release. Abnormally high NO levels are generally regarded as a key marker reflecting the progression of inflammatory reactions [19,20]. The CCK-8 results indicated that RAW 264.7 cell viability remained largely unchanged after treatment with CMR, CML, CMB, or CMF at the selected concentrations (Figure 6A). Compared with untreated cells, LPS challenge caused a pronounced elevation in NO levels (*p* < 0.001), demonstrating the successful induction of an inflammatory response. Further analysis revealed that extracts from all CM parts progressively reduced LPS-triggered NO release as the extract concentration increased (*p* < 0.05) (Figure 6B), indicating that these samples possessed certain anti-inflammatory potential. Among them, CML exhibited the lowest IC_50_ value (165.43 ± 24.14 μg/mL), indicating the strongest inhibitory capacity against NO production, followed by CMB (225.27 ± 18.41 μg/mL), CMR (254.00 ± 38.01 μg/mL), and CMF (257.43 ± 19.26 μg/mL). Consistent with the inhibitory effect on NO production, the ELISA data further demonstrated that CML markedly reduced the release of TNF-α, IL-6, and IL-1β in LPS-treated cells (Figure 7A–C). Compared with extracts from the other CM parts, CML showed stronger suppressive effects on these inflammatory cytokines, supporting its potential anti-inflammatory activity.

### 3.4. Correlation Analysis: Associations Between Metabolites and Bioactivities

**Figure 8:**
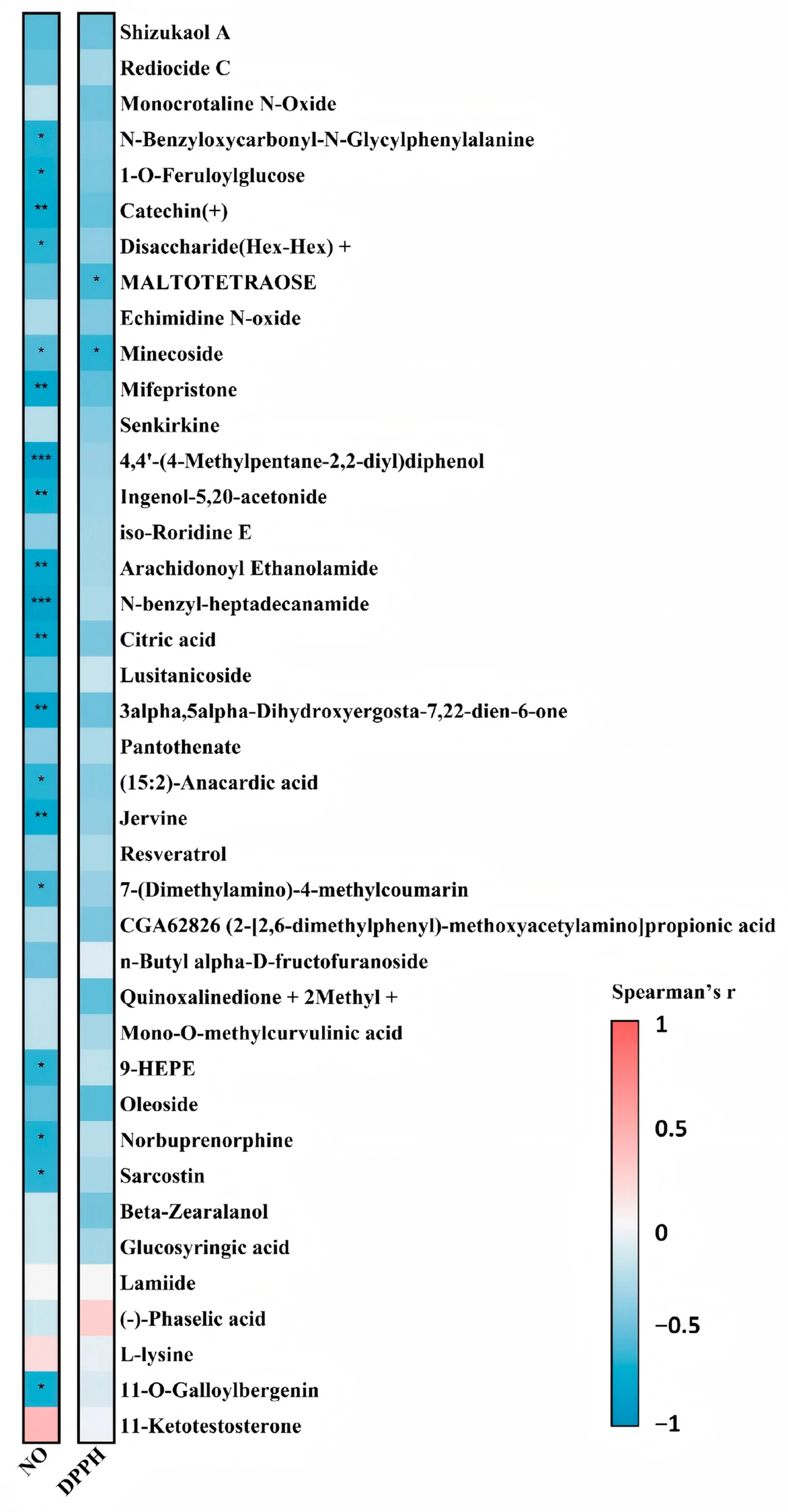
Spearman correlation heatmap of differential metabolites with IC_50_ values for DPPH scavenging and NO inhibition. Negative *r* (blue) indicates association with higher activity. * *p* < 0.05, ** *p* < 0.01, and *** *p* < 0.001.

To screen for small-molecule metabolites that may contribute to the bioactivities of CM extracts, Spearman rank analysis was applied to relate differential metabolite abundances to the IC_50_ values for DPPH radical scavenging and NO inhibition (Figure 8). The results showed that the feature putatively annotated as minecoside was negatively correlated with both the IC_50_ value for NO inhibition (*r* = −0.594, *p* = 0.042) and the IC_50_ value for DPPH radical scavenging (*r* = −0.664, *p* = 0.018), suggesting that it may be associated with differences in antioxidant and anti-inflammatory activities among different CMs. Maltotetraose was mainly negatively correlated with the IC_50_ value for DPPH radical scavenging (*r* = −0.620, *p* = 0.031), suggesting that it may be related to differences in antioxidant activity. In addition, metabolites such as (+)-catechin, arachidonoyl ethanolamide, and citric acid showed significant negative correlations with the IC_50_ value for NO inhibition (*p* < 0.01), suggesting that these compounds may be important candidate constituents associated with the anti-inflammatory activity of the extracts. Collectively, these findings suggest that the functional differences among CMs are not attributable to a single compound, but are more likely driven by the coordinated actions of diverse metabolites.

## 4. Discussion

In this study, an integrated approach combining UPLC-MS/MS-based untargeted metabolomics combined with in vitro antioxidant and anti-inflammatory assays was used to systematically compare the small-molecule metabolite compositions and differences in bioactivities among four CMs, namely CMF, CML, CMR, and CMB. The results demonstrated pronounced tissue specificity in the differential metabolites among the four CMs, accompanied by substantial variation in the antioxidant and anti-inflammatory effects of their extracts. These findings indicate that CML and CMR exhibited relatively stronger activities in the in vitro antioxidant or anti-inflammatory assays, suggesting that these non-fruit parts have potential for further development and in-depth investigation.

Regarding metabolite composition, the comparative analysis yielded 826 differential metabolites across the four CMs. Among them, prenol lipids accounted for the largest proportion (20.2%), followed by organooxygen compounds (10.9%) and flavonoids (10.0%). This composition is highly consistent with the chemical characteristics of plants in the genus *Citrus* of the family Rutaceae [21]. Notably, relatively fewer differential metabolites were found to be specifically enriched in CMF, whereas CML showed a richer profile of differential metabolites, especially with the relative enrichment of several metabolite classes: terpenoids (forskolin and shizukaol A), flavonoid and polyphenolic compounds (kuwanon G), coumarin glycosides (fraxidin glucoside), and unsaturated fatty acids (linolenic acid). This result does not negate the value of the fruit as the traditional medicinal part, but rather suggests that non-fruit parts may also possess noteworthy chemical research value. From the perspective of plant physiological functions, leaves are the major organs for photosynthesis and responses to environmental stimuli. The relative enrichment of terpenoids and flavonoids in leaves may be involved in photoprotection, antioxidant defense, and responses to pathogen stress [22,23]. Branches perform transport, support, and storage functions, and the relative enrichment of saccharides, such as amylopectin and isomaltotriose, and structural lipids, such as 12-hydroxyoctadecanoic acid, may be associated with energy storage and the maintenance of cell wall structural stability [24]. Roots are closely related to water absorption, nitrogen transport, and stress adaptation. The presence of nitrogen-containing compounds, such as L-asparagine, and oxidized fatty acids, such as FA 18:2+2O, may reflect their roles in nitrogen transport and oxidative stress responses [25,26,27,28]. Therefore, the metabolic differences among different CMs are not merely reflected in metabolite composition, but may also be closely associated with organ functional differentiation and the observed differences in bioactivity.

KEGG enrichment analysis indicated that the metabolic divergence among the four CMs extended beyond typical secondary metabolites, including flavonoids and coumarins. Several pathways related to amino acid biosynthesis and metabolism were significantly enriched, such as arginine biosynthesis, branched-chain amino acid biosynthesis, taurine and hypotaurine metabolism, β-alanine metabolism, and phenylalanine metabolism. These results suggest substantial differences in amino acid metabolism, nitrogen utilization, and other primary metabolic processes across different CMs. First, in plants, amino acids function as fundamental precursors for protein formation while also contributing to nitrogen reserve, carbon–nitrogen homeostasis, and stress adaptation [29,30,31]. Core metabolites such as L-glutamic acid, L-valine, γ-aminobutyric acid, L-asparagine, and citric acid were involved in multiple pathways, indicating that the metabolic differences among different CMs may originate from the overall remodeling of primary metabolic networks. Second, phenylalanine-derived pathways provide important precursors for a range of bioactive compounds, including flavonoid compounds, phenolic acid derivatives, and coumarin-type metabolites. The enrichment of this pathway further reflects the metabolic coordination between amino acid-related processes and the accumulation of secondary metabolites [32,33]. Therefore, the differences in bioactivities among different CMs may not be determined by a single class of compounds, but rather by the combined effects of multiple metabolic modules, including amino acid metabolism, lipid metabolism, phenylalanine metabolism, and flavonoid metabolism. Moreover, enrichment was also observed in lipid-related pathways, particularly those involving unsaturated fatty acid formation and the metabolism of α-linolenic and linoleic acids. Jasmonic acid appeared in both α-linolenic acid metabolism and plant hormone signal transduction, indicating that fatty acid-derived signals may participate in the metabolic differentiation of different CMs. Jasmonic acid and related lipid-derived signals are generally associated with plant defense responses, oxidative stress, and the regulation of secondary metabolism [34,35]. This suggests that the metabolic differences among different CMs may not only reflect differences in static chemical composition, but also reflect differences in metabolic regulation among different organs during defense responses and environmental adaptation.

Among the four CM extracts, CML and CMR displayed superior antioxidant performance, as reflected by their higher scavenging capacities toward DPPH and ABTS radicals. For DPPH radicals, CML and CMR produced similar IC_50_ values of 0.65 ± 0.08 mg/mL and 0.69 ± 0.05 mg/mL, respectively, indicating that CML possessed only slightly higher activity than CMR. In contrast, in the ABTS assay, CMR showed slightly stronger activity than CML. Taken together, both CML and CMR exhibited good in vitro antioxidant potential. It is worth noting that the trends observed in the DPPH and ABTS assays were not entirely identical, possibly because these two methods differ in radical type, reaction medium, and detection principle [36,37]. The DPPH radical assay is mainly suitable for evaluating the radical scavenging capacity of samples in organic or relatively hydrophobic systems, and the reaction process is more easily affected by steric hindrance and the hydrogen atom-donating ability of compounds. In contrast, the ABTS radical cation assay is applicable to compounds with a broader polarity range and better reflects electron transfer-related antioxidant capacity in aqueous environments [38,39]. Therefore, the slightly different activity rankings of CML and CMR in the two models suggest that their antioxidant constituents may differ in polarity, hydrogen atom-donating ability, electron transfer capacity, and spatial structure. This result also indicates that a single antioxidant model is insufficient to comprehensively evaluate the antioxidant capacity of natural products, highlighting the necessity of combined evaluation using multiple methods.

Following LPS stimulation of RAW 264.7 macrophages, the tested CM extracts showed varying degrees of suppression on NO generation. Among them, CML showed the strongest inhibitory effect on NO production, with an IC_50_ value of 165.43 ± 24.14 μg/mL, followed by CMB (225.27 ± 18.41 μg/mL), CMR (254.00 ± 38.01 μg/mL), and CMF (257.43 ± 19.26 μg/mL). Further ELISA measurements showed that CML effectively suppressed the production of TNF-α, IL-6, and IL-1β, suggesting that CML extract possesses notable anti-inflammatory activity in vitro. Oxidative stress is closely associated with inflammatory responses, and the anti-inflammatory activity of natural products may, to some extent, be related to their antioxidant capacity [40,41]. However, in this study, the ranking of anti-inflammatory activity was not completely consistent with that of antioxidant activity, suggesting that the anti-inflammatory effects were not entirely determined by total antioxidant capacity. In addition to their antioxidant contributions, specific bioactive metabolites may participate in the regulation of inflammatory responses through pathways associated with NO generation, cytokine production, and inflammatory signal transduction. Therefore, the anti-inflammatory potential of different CMs may result from the combined effects of antioxidant activity and characteristic metabolites, rather than being driven solely by total antioxidant capacity. These candidate metabolites may contribute to the anti-inflammatory effects of CMs through regulation of inflammation-related signaling pathways. Previous studies have suggested that finger citron extracts may attenuate inflammatory responses through modulation of NF-κB and MAPK signaling pathways, accompanied by reduced production of inflammatory mediators, including NO, TNF-α, and IL-6 [42]. In addition, finger citron essential oil and its constituents have been reported to inhibit JNK, ERK, and NF-κB activation in LPS-stimulated macrophages [43]. Therefore, the stronger anti-inflammatory activity observed in CML may be partially associated with the enrichment of flavonoids, phenolic compounds, and other bioactive metabolites identified in this organ. However, further molecular studies are required to confirm the specific targets and signaling pathways involved. In addition, although the present study demonstrated the anti-inflammatory potential of different CMs, the lack of a pharmacological anti-inflammatory positive control limited direct comparison with established anti-inflammatory agents. Moreover, because CMs contain multiple bioactive constituents, their overall pharmacological effects may differ from those of individual purified compounds. Future studies incorporating standard anti-inflammatory compounds as positive controls are needed to further evaluate the pharmacological potency of CMs.

To identify candidate metabolites contributing to the variation in CM bioactivities, Spearman’s rank method was used to link differential metabolite abundance with IC_50_ values related to DPPH radical scavenging and NO inhibition. We found that minecoside was significantly negatively correlated with both the IC_50_ value for NO inhibition (*r* = −0.594) and the IC_50_ value for DPPH radical scavenging (*r* = −0.664), suggesting that it may be one of the candidate metabolites associated with both antioxidant and anti-inflammatory activities. Existing evidence indicates that minecoside can attenuate inflammatory responses by blocking JAK/STAT3 signaling and lowering the production of IL-1β, TNF-α, and COX-2 [44,45]. In addition, this compound shows peroxyl radical-scavenging capacity, with a reported ORAC value of 1.23 μmol TE/μmol [46]. Maltotetraose showed a significant negative correlation with the IC_50_ value for DPPH radical scavenging, indicating its potential relevance to antioxidant activity. Previous research has shown that maltotetraose from total oligosaccharides of black ginseng can ameliorate oxidative stress injury by activating the Keap1/Nrf2 pathway [47]. In addition, several metabolites were significantly associated with anti-inflammatory activity. For example, (+)-catechin can exert both anti-inflammatory and antioxidant effects by activating the Nrf2 pathway and blocking the TLR4/MyD88/NF-κB pathway [48,49]. Arachidonoyl ethanolamide (AEA) can regulate NO levels in Müller glial cells and modulate COX-2, PGE_2_, and IL-6 in RAW 264.7 cells, thereby exerting anti-inflammatory activity [50,51]. The candidate metabolites screened in this study, such as minecoside, (+)-catechin, and maltotetraose, may provide directions for subsequent research. In the future, targeted quantification, activity evaluation of isolated compounds, component removal/add-back experiments, and validation of cellular signaling pathways could further clarify the contributions of these candidate metabolites to the antioxidant and anti-inflammatory activities of CM. However, correlation analysis can only provide clues regarding candidate bioactive constituents. Further investigations should focus on measuring total flavonoid and phenolic levels, quantifying key individual compounds, and confirming the biological activities of selected constituents. Targeted metabolomics approaches combined with authentic standard confirmation will be necessary to further verify the identities, abundance changes, and potential biological roles of these candidate metabolites. Moreover, inflammatory pathway verification and in vivo safety studies will provide a more comprehensive understanding of how chemical profiles are associated with the antioxidant and anti-inflammatory properties of CMs. Because evaporation and reconstitution may affect the recovery of volatile or labile constituents, the metabolomic profiles reported here represent the nonvolatile small-molecule fraction recovered under the applied sample-preparation conditions rather than the complete composition of the original ethanol extracts.

From the viewpoint of comprehensive resource utilization, CML appeared to be a promising non-fruit part of CM. It contained a relatively diverse set of differential metabolites and showed favorable bioactivities in both radical-quenching tests and the LPS-stimulated RAW 264.7 macrophage system. Considering that leaves are relatively easy to obtain during cultivation and pruning, and that their collection generally does not damage the main body of the plant, CML has good feasibility for further development as a by-product resource of CM. Although the present study demonstrated promising bioactivities in vitro, these effects and the bioavailability of the active constituents have not yet been confirmed in animal or other in vivo systems. Further in vivo studies, together with safety assessments, are therefore required. It is worth noting that the profiles of plant secondary metabolites are shaped by multiple factors, including cultivar, geographical origin, harvest period, growth stage, and environmental conditions. Although the tissue-specific metabolic differences observed in the present study are likely associated with intrinsic metabolic characteristics of different CM tissues, the potential influence of environmental factors cannot be completely excluded. Therefore, future studies should further compare the composition of major active constituents and the differences in antioxidant and anti-inflammatory activities of CML from different seasons and geographical origins, so as to clarify its quality stability, suitable harvest period, and basis for standardized utilization. In contrast, CMR showed antioxidant capacity comparable to that of CML, indicating that it also has certain research potential. However, root harvesting may affect plant growth and resource sustainability; therefore, its utilization should be comprehensively evaluated in combination with cultivation renewal, sources of processing by-products, and sustainable harvesting strategies.

## 5. Conclusions

In summary, this study demonstrated that different CMs exhibited distinct tissue-specific metabolic profiles, and that CML and CMR showed relatively higher antioxidant and anti-inflammatory activities in vitro. Among them, CML exhibited a relatively diverse profile of differential metabolites and favorable bioactivities under the tested experimental conditions, suggesting its potential value for further investigation as a non-fruit resource of CM. The candidate metabolites identified by correlation analysis may provide directions for subsequent studies on bioactive constituents and mechanisms. Overall, this study provides metabolomic and in vitro bioactivity evidence supporting the comprehensive utilization of non-fruit parts of CM. However, the biological functions of these candidate metabolites and the practical application potential of CM extracts require further validation through targeted quantification, authentic standard confirmation, isolated compound evaluation, and in vivo studies.

## Data Availability

The original contributions presented in the study are included in the article/Supplementary Materials; further inquiries can be directed to the corresponding authors. The metabolomic datasets generated in this study have been deposited in the MetaboLights repository under accession number MTBLS15148. The dataset is currently under review and will be publicly accessible upon publication.

## Supplementary Materials

The following supporting information can be downloaded at https://www.mdpi.com/article/10.3390/metabo16080555/s1, Table S1. Extraction yields of different parts of *Citrus medica* L. var. *sarcodactylis*; Table S2. UPLC–MS/MS operating conditions; Table S3. Data-processing and differential metabolite screening parameters; Table S4. Experimental conditions for DPPH and ABTS radical scavenging assays; Table S5. Experimental conditions for cell-based anti-inflammatory assays; Table S6. List of the 826 differential metabolite features with their putative annotations and relative abundances in different CMs; Table S7. Validation parameters of the PLS-DA model for CMs; Figure S1. Permutation test validation of the PLS-DA model for discrimination of CMs.
