# Supplementary material for: Untargeted Metabolomics Reveals Organ-Specific Metabolites Associated with Antioxidant and Anti-Inflammatory Activities in Finger Citron (Citrus medica L. var. sarcodactylis)
Source: Metabolites. 2026 Aug 6;16(8):555. doi: 10.3390/metabo16080555 (PMC13515510; doi:10.3390/metabo16080555)
Supplement: Supplementary file 1 [file metabolites-16-00555-s001.zip › Supplementary Materials.pdf]

Table S1 Extraction yields of different parts of *Citrus medica* L. var. *sarcodactylis*.

CM part	Extraction yield (%)
CMF	30.20
CML	13.64
CMR	11.80
CMB	6.46

Table S2. UPLC–MS/MS operating conditions.

Chromatographic conditions: Separation was performed on an ACQUITY UPLC HSS T3 column (100 mm × 2.1 mm, 1.8 μm; Waters, Milford, MA, USA). The column temperature, flow rate, and injection volume were set at 40 °C, 0.3 mL/min, and 2 μL, respectively. Mobile phase A was water containing 0.1% formic acid, and mobile phase B was acetonitrile containing 0.1% formic acid. The gradient program was as follows: 0–1 min, 0% B; 1–12 min, 0–95% B; 12–13 min, 95% B; 13.0–13.1 min, 95–0% B; and 13.1–17.0 min, 0% B.

Mass spectrometric conditions: MS detection was carried out using a Q Exactive HF-X Orbitrap MS/MS platform equipped with a heated electrospray ionization (HESI) source (Thermo Fisher Scientific, Waltham, MA, USA). The system was operated in Full MS/dd-MS2 mode with polarity switching between positive and negative ion modes. The source settings were as follows: sheath gas, 40 arb. units; auxiliary gas, 10 arb. units; spray voltage, +3000 V/−2800 V; ion transfer tube temperature, 320 °C; and auxiliary gas heater temperature, 350 °C. The full-scan range was m/z 70–1050, and the resolutions were 70,000 for MS1 and 17,500 for MS2.

Table S3. Data-processing, metabolite-annotation, and differential-metabolite screening parameters.

**Category**	**Parameters**

**Raw data acquisition software**	Xcalibur software, version 4.1 (Thermo Fisher Scientific, Waltham, MA, USA)
**Data preprocessing software**	Progenesis QI software, version 3.0.7927 (Waters Corporation, Milford, MA, USA)
**Data preprocessing procedures**	Baseline filtering, peak detection, peak matching, retention-time correction, peak alignment, and generation of the peak-intensity matrix
**Feature filtering and normalization**	Features with zero values in more than 80% of all samples were removed. Remaining peak-intensity data were normalized using the MetNormalizer package in R
**Quality control assessment**	QC samples were used to evaluate analytical stability. Features with QC relative standard deviation (RSD) >30% were excluded
**Metabolite annotation database**	HMDB and METLIN databases
**Annotation strategy**	Metabolite features with available MS/MS spectra were putatively annotated by integrating accurate molecular mass and MS/MS fragmentation patterns against HMDB and METLIN databases.
**Mass accuracy criterion**	Precursor molecular mass error within 10 ppm
**MS/MS spectral similarity evaluation**	Library-matching score ranging from 0 to 100; higher scores indicate greater spectral similarity and higher annotation confidence
**Secondary databases for metabolite information**	HMDB, KEGG Compound (public database), and LIPID MAPS

**integration and pathway annotation**	
**Database applications**	Metabolite information integration, KEGG identifier assignment, chemical classification, and pathway annotation
**KEGG pathway annotation**	Pathway annotation was performed by matching annotated metabolites with identifiers from the publicly available KEGG Compound database
**Multivariate statistical analysis**	Partial least-squares discriminant analysis (PLS-DA) was performed using the ropls package, version 1.32.0, in R.
**Statistical analysis**	One-way analysis of variance (ANOVA), followed by Tukey's multiple-comparison test
**Differential metabolite screening criteria**	Differential metabolites were screened by integrating VIP > 1, $p < 0.05$, and FC > 1.5 or FC < 0.67

Table S4. Experimental conditions for DPPH and ABTS radical scavenging assays.

For the DPPH assay, 100 μL of each extract solution at different concentrations was mixed with 100 μL of 0.2 mM DPPH solution. The mixture was kept in the dark at room temperature for 30 min, and the absorbance was then measured at 517 nm.

For the ABTS assay, equal volumes of 7 mM ABTS solution and 2.45 mM potassium persulfate solution were combined and allowed to react in the dark at room temperature for 12 h to generate ABTS•+. The resulting solution was diluted with ultrapure water until the absorbance reached 0.70 ± 0.02 at 734 nm. Subsequently, 180 μL of ABTS working solution was mixed with 20 μL of each extract solution. After 5 min of incubation in the dark at room temperature, the absorbance was recorded at 734 nm.

BHT was used as the positive control in both assays, and all measurements were performed in triplicate.

Table S5. Experimental conditions for cell-based anti-inflammatory assays.

For cell treatment, the 70% ethanol extracts were concentrated under reduced pressure and freeze-dried. The resulting dry extracts were dissolved in DMSO and diluted with complete culture medium to the required concentrations. The final DMSO concentration did not exceed 0.1% (v/v). Before cell treatment, all extract solutions were passed through a 0.22 μm sterile membrane.

RAW 264.7 murine macrophages were cultured in high-glucose DMEM supplemented with 10% FBS and 1% penicillin–streptomycin solution at 37 °C in a humidified atmosphere containing 5% CO_2. To construct the inflammatory model, cells were stimulated with LPS at 1 μg/mL for 24 h. Untreated cells served as the control group, and cells treated only with LPS were used as the model group.For the CCK-8 assay, cells in the logarithmic growth phase were seeded into 96-well plates at 1×10^4 cells/well and allowed to attach overnight. The medium was replaced with 100 μL of complete medium containing the extracts at 0, 25, 50, 100, 200, or 400 μg/mL, expressed as dry extract equivalents. After 24 h of incubation, the medium was removed, and 100 μL of fresh medium containing 10% (v/v) CCK-8 reagent was added to each well. Following incubation at 37 °C for 1 h, absorbance was recorded at 450 nm using a microplate reader. Cell viability was calculated according to a previously reported method [49]. For NO determination, cells were treated as described above, and culture supernatants were collected after 24 h. Nitrite accumulation, as an indicator of NO production, was measured using Griess reagent. Absorbance was detected at 540 nm, and nitrite concentrations were calculated from a sodium nitrite standard curve.For cytokine analysis, the concentrations of TNF-α, IL-6, and IL-1β in the collected culture supernatants were measured using ELISA kits according to the manufacturers' instructions. Absorbance was measured at 450 nm, and cytokine levels were calculated based on the corresponding standard curves. All measurements were performed in triplicate.

Table S6. List of the 826 differential metabolite features with their putative annotations and relative abundances in different CMs.

Table S7. Validation parameters of the PLS-DA model for CMs.

Component	R^2X	R^2X(cum)	R^2Y	R^2Y(cum)	Q^2	Q^2(cum)
p1	0.323	0.323	0.330	0.330	0.106	0.106
p2	0.176	0.499	0.324	0.654	0.297	0.372

Model: CMR vs CMB vs CML vs CMF

Permutation test: 200 permutations

R^2X represents the proportion of variance in the X matrix explained by each predictive component. R^2X(cum) represents the cumulative proportion of X variance explained by the model. R^2Y represents the proportion of variance in the Y matrix explained by each predictive component, and R^2Y(cum) represents the cumulative explanatory ability of the model. Q^2 represents the predictive ability of each component estimated by cross-validation, while Q^2(cum) represents the cumulative predictive ability of the PLS-DA model. The reliability of the model was further evaluated using a 200-permutation test.

Figure S1. Permutation test validation of the PLS-DA model for discrimination of CMs.

The reliability of the PLS-DA model was evaluated using a 200-permutation test. The original PLS-DA model was compared with models generated after random permutation of class labels. The lower Q^2 values obtained from the permuted models and the negative intercept of the Q^2 regression line indicated that the PLS-DA model was not overfitted.

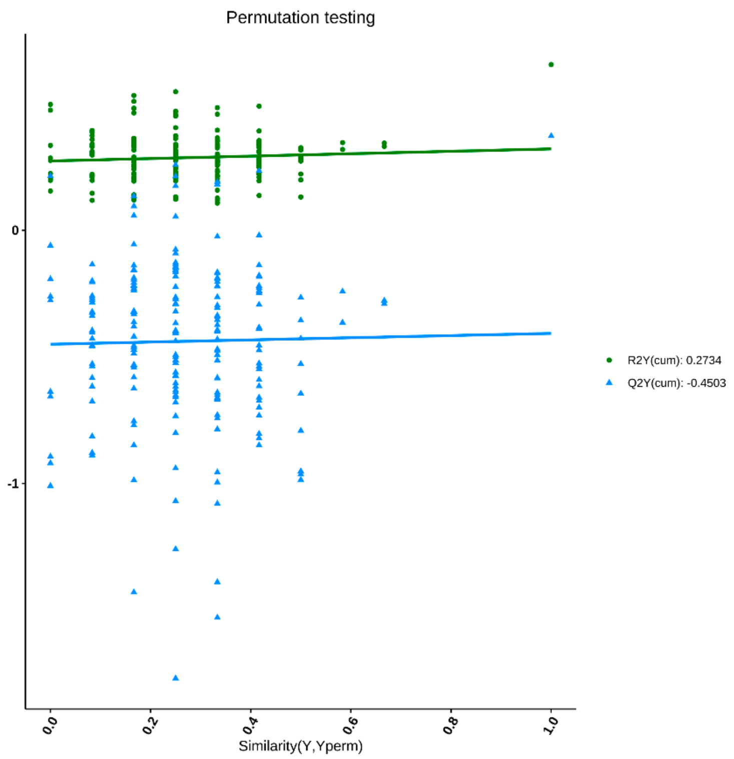
Permutation testing
0
-1
0.0
0.2
0.4
0.6
0.8
1.0
Similarity(Y,Yperm)
R2Y(cum): 0.2734
Q2Y(cum): -0.4503
